# Predictors of Poststroke Aphasia Recovery: A Systematic Review-Informed Individual Participant Data Meta-Analysis

**DOI:** 10.1161/STROKEAHA.120.031162

**Published:** 2021-03-15

**Authors:** Marian C. Brady

**Affiliations:** Nursing, Midwifery and Allied Health Professions Research Unit, Glasgow Caledonian University, United Kingdom.

**Keywords:** aphasia, comprehension, demography, language, survivor

## Abstract

**Background and Purpose:**

The factors associated with recovery of language domains after stroke remain uncertain. We described recovery of overall-language-ability, auditory comprehension, naming, and functional-communication across participants’ age, sex, and aphasia chronicity in a large, multilingual, international aphasia dataset.

**Methods:**

Individual participant data meta-analysis of systematically sourced aphasia datasets described overall-language ability using the Western Aphasia Battery Aphasia-Quotient; auditory comprehension by Aachen Aphasia Test (AAT) Token Test; naming by Boston Naming Test and functional-communication by AAT Spontaneous-Speech Communication subscale. Multivariable analyses regressed absolute score-changes from baseline across language domains onto covariates identified a priori in randomized controlled trials and all study types. Change-from-baseline scores were presented as estimates of means and 95% CIs. Heterogeneity was described using relative variance. Risk of bias was considered at dataset and meta-analysis level.

**Results:**

Assessments at baseline (median=43.6 weeks poststroke; interquartile range [4–165.1]) and first-follow-up (median=10 weeks from baseline; interquartile range [3–26]) were available for n=943 on overall-language ability, n=1056 on auditory comprehension, n=791 on naming and n=974 on functional-communication. Younger age (<55 years, +15.4 Western Aphasia Battery Aphasia-Quotient points [CI, 10.0–20.9], +6.1 correct on AAT Token Test [CI, 3.2–8.9]; +9.3 Boston Naming Test points [CI, 4.7–13.9]; +0.8 AAT Spontaneous-Speech Communication subscale points [CI, 0.5–1.0]) and enrollment <1 month post-onset (+19.1 Western Aphasia Battery Aphasia-Quotient points [CI, 13.9–24.4]; +5.3 correct on AAT Token Test [CI, 1.7–8.8]; +11.1 Boston Naming Test points [CI, 5.7–16.5]; and +1.1 AAT Spontaneous-Speech Communication subscale point [CI, 0.7–1.4]) conferred the greatest absolute change-from-baseline across each language domain. Improvements in language scores from baseline diminished with increasing age and aphasia chronicity. Data exhibited no significant statistical heterogeneity. Risk-of-bias was low to moderate-low.

**Conclusions:**

Earlier intervention for poststroke aphasia was crucial to maximize language recovery across a range of language domains, although recovery continued to be observed to a lesser extent beyond 6 months poststroke.

## Abstract 2

Supplemental Digital Content is available in the text.

Approximately one-third of the 25.7 million stroke survivors^1^ worldwide experience aphasia,^2^ affecting spoken language, auditory and reading comprehension, writing, and everyday communication. Aphasia is associated with poorer performance on functional recovery^3^ activities of daily living,^4^ and emotional well-being after stroke.^5^ Aphasia also affects hospital discharge destination^6^ and the likelihood of successful return to work. Long-term language impairment affects 61% of stroke survivors’ communication at 1 year after onset.^7^ While spontaneous recovery seems limited after that point^8,9^ focused therapeutic intervention may benefit people with chronic aphasia.^10^ Evidence is needed to clarify the nature and role of demography, stroke, and aphasia profiles on language recovery after stroke.

Clinical guidelines^11^ recommend communication of realistic prognoses for recovery. However, accurate prognostication based on existing evidence is challenging,^12^ due to use of single-center studies offering small sample sizes^13^ and investigating single language domains,^14^ thereby limiting information on the recovery potential across the spectrum of impaired language domains and demography for people with aphasia.

Severity of aphasia at onset has been linked with recovery^15,16^ but is often categorized in broad terms (eg, mild/moderate/severe)^7^ or as an item within a global stroke severity measure,^17^ lacking sensitivity to provide clinically meaningful indications of recovery potential across the range of affected language domains. Previous studies examining aphasia profiles and recovery have been limited to literature reviews and aggregated summary data,^18^ retrospective review of hospital records,^19,20^ nonstandardized determination of aphasia, or aggregation within systematic reviews,^13^ often using English-speaking datasets, uncontrolled and nonrandomized studies.^21^ Associations between age, sex, and language recovery have been suggested, but the nature of that interaction remains unclear.^7,22^ The degree of recovery from aphasia across the poststroke trajectory is also uncertain, despite variable evidence for early intervention,^23^ with some suggesting intervention should be delayed.^24^

The recovery potential across the spectrum of people with aphasia requires analysis of large samples reflecting a range of demographic variables, across multiple geographic centers, with detailed assessment of language domains, using standardized methods of data collection, within a clinically relevant time-frame. A pragmatic approach using existing datasets may inform clinical insights and direct future research. Systematic review-based Individual Participant Data (IPD) meta-analysis allows for individual representation and exploration of missing data, as well as individual adjustment for prognostic factors on a larger scale. Examining IPD from several studies facilitates greater participant representation,^25^ enables subgroup analyses, and can synthesize smaller and disparate datasets at IPD level without bias that might be introduced through the use of single-center meta-analyses.

We conducted an IPD meta-analysis using a rigorous, systematically collated international aphasia research database to identify demographic, stroke- and language-related factors associated with aphasia recovery.

## Methods

### Ethics, Protocol, Registration, and Guidelines

The Rehabilitation and Recovery of People With Aphasia After Stroke Project collated data from completed aphasia studies (University ethical approval: HLS/NCH/15/09; PROSPERO; CRD42018110947; IRAS; database ID 179505). The protocol for development of this database including search strategy and eligibility criteria was published elsewhere^26^; methods and findings are reported according to the PRISMA-IPD guidelines.

### Data Availability

Where contributors have given permission, fully anonymized datasets will be made available to the wider research community through the Collaboration of Aphasia Trialists (https://www.aphasiatrials.org/aphasia-dataset/) from December 2020.

### Procedures

**Figure 1.**
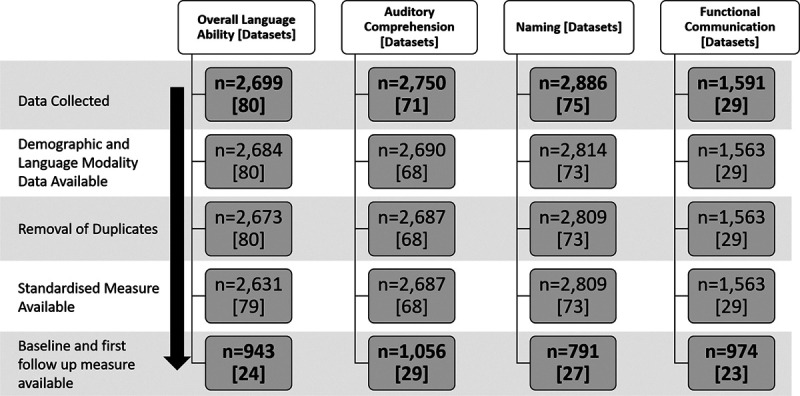
**Flowchart of individual participant data and dataset inclusion.** n=individual participant data.

We systematically identified datasets with at least 10 people with poststroke aphasia, documented language assessments and time since index stroke from MEDLINE, EMBASE, CINAHL, LLBA, and SpeechBITE (inception to September 2015) supplemented by a search of trial registrations to identify emerging datasets beyond this period. Building upon previous aggregated trial data syntheses,^27^ we extracted anonymized IPD on demography (age, sex, handedness, language of assessment, education level, socioeconomic status), time from stroke onset to inclusion in the primary research study, type of stroke, study design, language outcome data (overall-language-ability, auditory comprehension, naming, other spoken language, reading comprehension, writing, and functional-communication), and timing of language assessment. Assessment instruments were categorized by the language domain measured; categorizations were reviewed, discussed, and accepted by the Rehabilitation and Recovery of People With Aphasia After Stroke Collaborators a priori. We retained complete IPD on language domain assessments where available at both baseline and first follow-up (Figure 1).

### Standardization of Outcomes

We converted IPD gathered on multiple assessment instruments into one standardized measurement for each language domain (see Table I in the Data Supplement) as previously reported.^26^ Data integrity was assessed by performing checks for ranges, missingness, and expected formats. Selection, detection, attrition, and reporting biases were assessed at study and database level.

Primary analysis was based on randomized controlled trial (RCT) IPD; secondary analyses included all study designs (RCT plus non-RCT group comparisons, cohort, case series and registry datasets). Analyses were specified a priori and comprised at least 2 source datasets. Our primary outcome was mean absolute change in language domain score from baseline to first follow-up. Secondary outcomes were absolute and relative proportions of change in language scores from baseline.

### Statistical Analysis

A one-stage meta-analysis approach combined available IPD from eligible datasets with analyses preserving the clustering of participants within each study and were generated using SAS/STAT software, Version 9.4 of the SAS System for Windows.

Where possible, we presented data according to whether the participant had access to speech and language therapy (SLT). People with aphasia following a first stroke, allocated to receive no study-mediated SLT, no standard care SLT, and who were enrolled within 15 days of stroke onset (reducing the possibility of unreported historical SLT exposure) were described as the No SLT group. People with aphasia who were exposed to any form of SLT (eg, as part of a study or standard SLT care during the study period) were the SLT group. A historical SLT group comprised people who received no study-mediated SLT intervention, no standard SLT care during the study period but who were enrolled after 15 days of onset and, unless otherwise stated, were therefore likely to have received SLT as part of standard care in the past.

Within each domain, we performed meta-analysis with fixed demographic and recovery effects and included study as a random effect; variance was also evaluated. The absolute change in language domain scores from baseline to first follow-up was regressed onto stroke, aphasia, and demographic covariates specified a priori (baseline language impairment score, age, sex, lesioned hemisphere, stroke type, handedness, language of data collection, and time since stroke). Only variables that were significant at univariable level (*P*<0.1) were included in the multivariable analysis. Each multivariable analysis included the study as a random covariate to account for possible variability in the outcome measure between studies, with the demographic variables modeled jointly. This allowed us to present each demographic variable as the independent effect following the inclusion of the other variables in the model and allowed us to avoid the effect of confounding. Results were presented as estimate mean change in absolute scores from baseline and 95% confidence limits.

We described recovery in the context of absolute and relative proportions of change from baseline. Absolute proportion of change was described as the difference between scores at baseline and first follow-up, expressed as a proportion of the maximum possible assessment instrument score 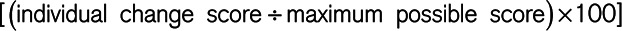. Relative proportion of change since baseline was described as the proportion of change in score at first follow up, relative to baseline score 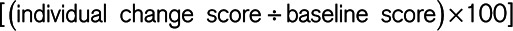. Absolute proportion has a ceiling of 100% and assumes that recovery scales are linear; that is, an improvement of 20% anywhere on the scale from a starting point of 0 to 80 is equivalent. Relative proportion is unbounded and emphasizes gains at the more severe end of the scale (eg, a patient recovering from 10% to 30% will improve by 200% on this measure, while a person recovering from 70% to 90% will improve by 29%).

We examined chronicity since stroke for each language domain across the following study entry points: 0 to 1, 1 to 3, 3 to 6, and >6 months. We accounted for the skewness of data for each language domain by presenting medians and interquartile ranges (IQRs).

## Results

We screened 5256 records. From 698 potentially eligible datasets (592 resulted in no data contribution: 193/592 were trial registration records, 318 received no responses, 78 had no available data and 3 declined to participate), 174 research studies were included (IPD=5928; 24.9% of potentially eligible datasets). We included IPD from 47 RCTs, 18 non-RCTs, 5 registries, and 104 case-series/cohort studies and extracted data on demography and language outcomes for analysis; median age was 63 (IQR, 53–72), 3407/5550 (61.4%) were male and median aphasia chronicity was 321 (IQR, 30–1156) days. Table II in the Data Supplement describes participant demographics across each language domain. Characteristics of included studies are reported elsewhere.^28^

Data checks revealed no clustering of transformed language domain scores that were generated from any single assessment instrument (Figure I in the Data Supplement). The transformed values were therefore considered to be accurate and valid. Data at baseline (median=43.6 weeks since stroke; IQR [4–165.1]) and first follow-up (median=10 weeks since baseline; IQR [3–26]) were available for overall-language-ability (n=943; 24 datasets, represented by the Western Aphasia Battery-AQ), auditory comprehension (n=1056; 29 datasets, represented by the Aachen Aphasia Test [AAT] Token Test), naming (n=791, 27 datasets, by Boston Naming Test), and functional-communication (n=974, 23 datasets, represented by the AAT Spontaneous-Speech Communication subscale, Figure 1). Data were extracted on other spoken language production (n=231, represented by the Porch Index of Communicative Ability), reading comprehension (n=219, represented by the Reading subtest of the Comprehensive Aphasia Test), and writing (n=253, represented by the Writing subtest of the Comprehensive Aphasia Test). Results for language production, reading comprehension, and writing are not presented as they did not meet the threshold for analysis defined a priori (at least 2 datasets), did not include RCT data, or contained too few subgroup data points for reliable analyses.

There were inadequate data on handedness (left-handed n=133; ambidextrous n=27) in combination with other key demographic variables to permit informative covariate-adjusted analyses. Socioeconomic status (n=175) and education level (n=3125) were reported using a range of nonaggregable formats (eg, multiple deprivation index, years of education and occupation). In combination with available outcome measurements, these resulted in very small strata within analyses and hindered meaningful covariate adjustment. Education, handedness, and socioeconomic status were therefore excluded as covariates from our analyses.

There were also insufficient language data on participants where we could be confident that had No SLT before or during the primary research (IPD=0 for overall-language-ability; IPD=15 for auditory comprehension; IPD=29 for naming). Data on participants in the historical SLT only group were limited to 2 RCTs (IPD=22) and 3 datasets in all study types (IPD=34); corresponding postintervention follow-up values were unavailable or did not meet our minimum eligibility criteria for analysis. Data are therefore presented on those who had access to SLT during the study intervention period.

### Predictors of Language Recovery

#### Overall-Language-Ability

Within the RCT datasets, the largest mean absolute change in overall-language-ability (presented as Western Aphasia Battery-AQ points) was seen in those aged <55 years (+15.4 points CI [10–20.9] IPD=136, 11 RCTs). Gains were also observed in those aged 56 to 65 years (+12.4points; IPD=141, 11 RCTs), 66 to 75 years (+11.5 points; IPD=96, 10 RCTs), and >75 years (+13.8 points; IPD=109; 7 RCTs; see Table III in the Data Supplement).

When examining aphasia chronicity, enrollment within 1 month of stroke was associated with greatest mean absolute change in overall-language-ability (+19.1 points on the, CI [13.9–24.4]; IPD=260, 8 RCTs). Gains were also significant for participants enrolled at later time points (1–3 months: +16.2 points [IPD=64, 6 RCTs]; 3–6 months: +9.6 points [IPD=16, 3 RCTs] and >6 months +8.2 points [IPD=142, 4 RCTs]).

Women experienced slightly greater gains in overall-language-ability (+14.3 points from baseline; 95% CI [9–19.5], IPD=206, 11 RCTs) compared with men (+12.3 points 95% CI [7.2–17.4]; IPD=276, 11 RCTs); however, score differences were not clinically meaningful. These observations were also consistent when analyzing data from all study types (see Table III in the Data Supplement).

#### Auditory Comprehension

Based on the analysis of RCT datasets, younger people experienced greater gains in auditory comprehension (presented as correct items on the AAT Token Test; <55 years: +6.1 correct; CI [3.2–8.9]; IPD=178, 16 RCTs) than older people. More correct responses were observed for enrollment within 1 month of stroke (+5.3 correct, 95% CI [1.7–8.8], IPD=139, 6 RCTs); gains were also evident >6 months post aphasia onset (+1.4 correct, 95% CI [−1.9 to 4.7] IPD=243, 9 RCTs). These observations were also consistent when analyzing data from all study types (Table IV in the Data Supplement).

#### Naming

Younger people (<55 years; IPD=103, 13 RCTs) experienced a gain of +9.3 points on the Boston Naming Test (CI [4.7–13.9]). Gains of +6.2 and +4.4 points were evident for people aged 66 to 75 years (IPD=97, 12 RCTs) and >75 years (IPD=61, 11 RCTs), respectively. When examining aphasia chronicity, the greatest gain was observed for enrollment within 1 month of stroke (+11.1 points, CI [5.7–16.5], IPD=129, 5 RCTs). Gains were also apparent in those enrolled at 1 to 3 months (+7.7 points; IPD=93, 8 RCTs), 3 to 6 months (+4.3 points; IPD=70, 6 RCTs), and >6 months (+4.1 points; IPD=93, 7 RCTs). These observations were also evident when analyzing all study types (Table V in the Data Supplement).

#### Functional Communication

Younger people (<55 years, IPD=147, 14 RCTs) experienced an absolute gain of +0.75 points on the AAT Spontaneous-Speech Communication subscale (CI [0.5–1]). Gains were also seen for those aged 56 to 65 years (+0.7 points; IPD=145, 13 RCTs), 66 to 75 years (+0.55 points; IPD=121, 14 RCTs), and >75 years (+0.65 points; IPD=119, 12 RCTs).

When examining chronicity, the greatest absolute gain was observed for enrollment within 1 month of stroke (+1-point, 95% CI [0.7–1.4]; IPD=232, 6 RCTs). Gains were also evident at later time points (1–3 months=+0.87 points [IPD=68, 5 RCTs]; 3–6 months=+0.4 points [IPD=62, 4 RCTs]; >6 months=+0.33 points [IPD=170, 7 RCTs]).

Women (IPD=236, 14 RCTs) experienced greater gains in functional-communication (+0.76 points [95% CI, 0.5–1]), compared with men (+0.57 points [95% CI, 0.3–0.8]; IPD=296, 14 RCTs). These observations were consistent with analysis of all study types (Table VI in the Data Supplement).

### Proportion of Recovery Across Each Language Domain

#### RCT Population

**Figure 2.**
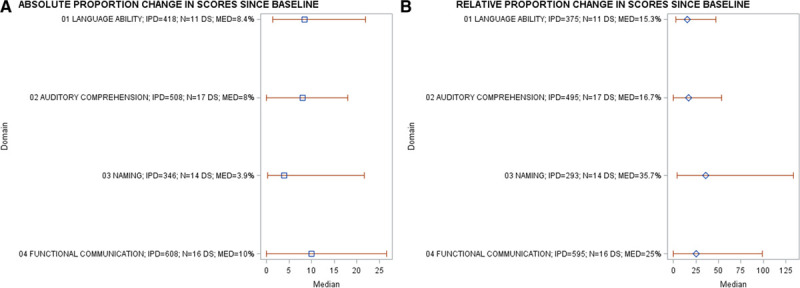
**Absolute and relative proportions of recovery across all language domains in the randomized controlled trial population.** IPD indicates individual participant data; MED, median proportion of recovery; and N, number of datasets.

We considered the absolute and relative proportions of change on each language domain score from baseline. The largest absolute proportion of change was observed on measures of functional-communication (median=10%; IQR [0%–26.6%]; 16 RCTs, 608 IPD) and overall-language-ability (median=8.4% IQR [1.3%–22%], 11 RCT, 418 IPD, Figure 2A), while the largest relative proportion of change was in naming (median=35.7%; IQR [4.2%–133.3%]; 14 RCTs, 293 IPD) and functional-communication (median=25% IQR [0%–98.9%], 16 RCTs, 595 IPD; Figure 2B).

**Figure 3.**
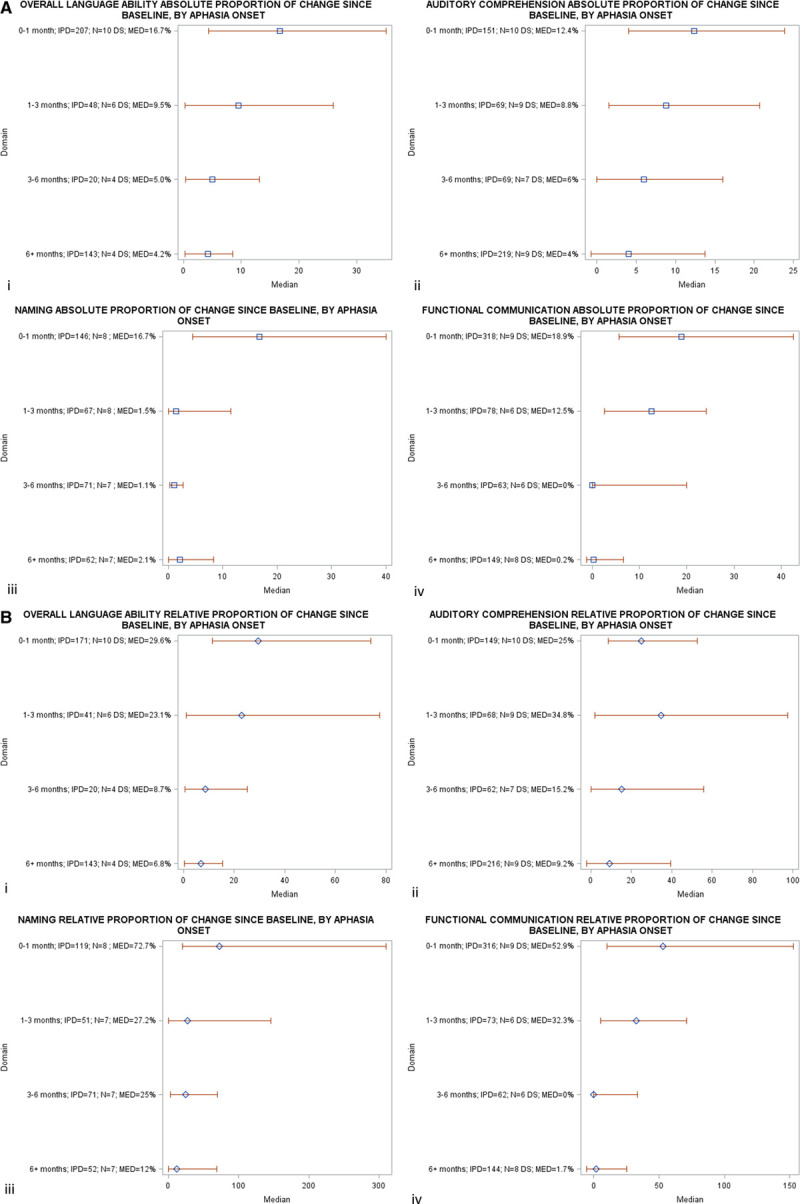
**A, Absolute proportion of recovery across all language domains, stratified by time since index stroke, in randomized controlled trial (RCT) populations.** **B**, Relative proportion of recovery across all language domains, stratified by time since index stroke, in RCT populations. DS indicates datasets; IPD, individual participant data; MED, median recovery; and N, number of datasets.

We observed a greater absolute (16.7% for overall-language-ability; IQR [4.3–35.1]; 12.4% for auditory comprehension; IQR [4–23.9]; 16.7% for naming; IQR [4.5–40]; and 18.9% for functional-communication; IQR [5.6–42.6] Figure 3A) and relative proportion of change (29.6% for overall-language-ability; IQR [11.3–73.9]; 72.7% for naming; IQR [20–310]; and 52.9% for functional-communication; [9.8–152.9] Figure 3B) for enrollment within 1 month of onset compared with other time windows poststroke.

#### All Study Designs

Data were available on overall-language-ability (IPD=788, 21 datasets); auditory comprehension (IPD=921, 27 datasets); naming (IPD=646, 24 datasets); and functional-communication (IPD=802, 22 datasets). We observed the largest absolute proportion of change in scores since baseline for functional-communication (9.5% IQR [0%–26.6%]) and overall-language-ability (6.8% IQR [0.3%–19.3%], Figure II in the Data Supplement), and the largest relative proportion of change for naming (IPD=582, 24 datasets; median=24.3% IRQ [0–115.4], see Figure II in the Data Supplement).

#### Heterogeneity and Bias

We found no evidence of significant statistical heterogeneity. Within all study types, most participant groups were comparable with respect to demographic, stroke, and aphasia severity variables at baseline, with baseline significant differences for subgroups only evident for age in 3/47 RCTs, sex in 1/47 RCTs, time since stroke in 2/47 RCTs, and language impairment in 1/47 RCTs. RCT datasets within Rehabilitation and Recovery of People With Aphasia After Stroke had a low risk of several sources of bias; most RCTs reported outcome assessor blinding (n=34/47; 75%). Attrition bias was low, and there was a moderate to low risk of selection bias, despite the use of predominantly research datasets. We considered the level of evidence available to be moderate because of potentially eligible datasets being identified in our systematic search but were not confirmed as eligible or contributed to Rehabilitation and Recovery of People With Aphasia After Stroke.^28^ Our analysis dataset showed no evidence of publication bias by sample size (*P*=0.77). Comparisons of missingness with the main demographic variables did not reveal any systematic relationships.

## Discussion

Our findings improve our understanding of poststroke language recovery, the domains that recover most, and the degree of recovery reported (in relative and absolute terms), and in which poststroke time windows the greatest recovery takes place. We observed the greatest improvement for enrollment within 1-month poststroke across all language domains. Improvements in mean absolute scores from baseline diminished with increasing time since stroke, yet still exceeded established group-level benchmarks of significant change for overall-language-ability (5.03 Western Aphasia Battery-AQ points) and naming (3.3 Boston Naming Test points).^29^ Relative and absolute proportions of change in scores across each language domain were typically the greatest within 1 month of stroke onset.

Our findings have important implications for the timing of SLT delivery; earlier intervention was associated with the greatest improvement across language domains. However, should early intervention be infeasible, for example, due to concurrent illness or inability to engage in rehabilitation, significant improvements were still observed beyond the acute period. We demonstrated clinical gains in the cohort who were enrolled beyond 6 months poststroke, which included those with a chronicity beyond 2 years. This population typically received usual care before study enrollment and still made clinical gains in language outcomes. In addition to greater improvements observed in the youngest population (<55 years) across all domains, we also observed substantial rehabilitation potential for elderly participants (>75 years), where gains of +13.8 Western Aphasia Battery-AQ and +4.4 Boston Naming Test points also exceeded the established group-level benchmarks of significant change for overall-language-ability and naming, respectively.^29^

We employed novel data transformation methods to standardize the language outcome data, and robust data synthesis methods including a systematic search for international and multilingual aphasia studies, including IPD from not only RCTs but also case-series/cohort studies, registries and non-RCTs. Inclusion of international, clinical datasets alongside research datasets enhanced the relevance of our data. We provided evidence primarily based on RCT sources and validated these observations in data from all study types. Importantly, collation and standardization of data from these sources permitted analysis of a much larger sample size and included a broader range of language domains than previously available. Our analyses preserved the clustering of participants within each trial.^30^ We reduced the risk of bias and increased applicability by preventing analyses to be undertaken on single datasets. Our operational definition of recovery was based on absolute proportion of change in scores from baseline, consistent with existing definitions.^15^

While some previous studies have described a beneficial impact of intervention within 3 months of onset, these analyses were based on data from English-language only, uncontrolled and nonrandomized studies.^21^ Other studies recommended delayed interventions^24,31^ or reported on SLT initiation after 12 weeks post-onset.^32^ Our study extends these findings, describing marked improvements in language outcomes for earlier initiation of therapy, compared with later initiation in a large, multilingual, international sample, and provides data across a range of affected language domains.

We were unable to account for spontaneous recovery in our study. Many potentially important covariates such as language stimulation in the living environment, other concomitant rehabilitation interventions, education level, initial stroke severity, mood disorders, co-existing cognitive impairments, and socioeconomic status were inconsistently available across the datasets, and therefore could not inform our planned analyses. Additionally, there were inadequate data on reading and writing assessments to permit examination of these factors. While we collated data from almost 6000 IPD, presence of single assessment time points, availability of language domain assessments, demographic and clinical factors only permitted analyses of samples ranging between 943 and 1056 IPD.

Nevertheless, our estimates and predictors of recovery are robust, use clinically meaningful assessment instruments, adjusting for critical participant confounders and are based on analyses of much larger sample sizes than previously examined.^33^ Our findings indicate a need for further investigation of therapy-associated recovery effects, and subgroup differences to ascertain which subpopulations may respond better to different types of intervention. Planned analyses will explore the associations between treatments, intensities, durations, dosages, and language outcomes.

## Acknowledgments

This project arose within the Tavistock Trust for Aphasia funded Collaboration of Aphasia Trialists.

## Sources of Funding

This project was funded by the National Institute for Health Research Health Services and Delivery Research (NIHR HS&DR project number 14/04/22) and the Tavistock Trust for Aphasia and will be published in full in the Health Services and Delivery Research Journal. Further information is available at www.journalslibrary.nihr.ac.u/programmes/hsdr/140422#/. The scientific content was reviewed and informed by discussion with the NIHR Complex Reviews Support Unit, also funded by the NIHR (project number 14/178/29). The views expressed are those of the author(s) and not necessarily those of the NIHR or the Department of Health and Social Care. The study also received infrastructural support from the Nursing, Midwifery and Allied Health Professions Research Unit (NMAHP Research Unit); NMAHP RU and MCB are funded by the Chief Scientist Office (CSO), Scottish Government Health and Social Care Directorates.

## Disclosures

Marian Brady received grants from Chief Scientist Office (CSO), Scottish Government Health and Social Care Directorates; EU Cooperation in Science and Technology (COST) funded Collaboration of Aphasia Scientists (IS1208 www.aphasiatrials.org); The Tavistock Trust for Aphasia during the conduct of the study; membership of the Royal College of Speech and Language Therapists. Data from the research of Audrey Bowen is included within the analyses in the RELEASE report. Her post at the University of Manchester is partly funded by research grants and personal awards from NIHR and Stroke Association. Caterina Breitenstein received grants from the German Federal Ministry of Education and Research (BMBF) during the conduct of the study. Erin Godecke received grants from Western Australian State Health Research Advisory Council (SHRAC) Research Translation Project Grants RSD-02720; 2008/2009 during the conduct of the study. Neil Hawkins received grants from National Institute for Health Research during the conduct of the study. Katerina Hilari received grants from The Stroke Association; European Social Fund and Greek National Strategic Reference Framework; The Tavistock Trust for Aphasia outside the submitted work. Petra Jaecks received PhD grant from Weidmüller Stiftung. Brian MacWhinney received grants from National Institutes of Health. Rebecca Marshall received grants from National Institute of Deafness and Other Communication Disorders, National Institutes of Health, during the conduct of the study. Rebecca Palmer received grants from NIHR senior clinical academic lectureship; NIHR HTA; Tavistock Trust for Aphasia outside the submitted work. Ilias Papathanasiou received funding from European Social Fund and Greek National Strategic Reference Framework. Jerzy Szaflarski received personal fees from SK Life Sciences; LivaNova, Inc, personal fees from Lundbeck; NeuroPace, Inc; Upsher-Smith Laboratories, Inc; grants and personal fees from SAGE Pharmaceuticals; UCB Pharma; grants from Biogen; Eisai, Inc, and other from GW Pharmaceuticals, outside the submitted work. Shirley Thomas received research grants from NIHR and The Stroke Association outside the submitted work. Ineke van der Meulen received grants from Stichting Rotterdams Kinderrevalidatiefonds Adriaanstichting, other from Stichting Afasie Nederland, other from Stichting Coolsingel, and other from Bohn Stafleu van Loghum during the conduct of the study. Linda Worrall received a grant from the National Health and Medical Research Council of Australia.

## Supplemental Materials

Tables I–VI

Figures I and II

## Supplementary Material



## Appendix

Myzoon Ali (Nursing, Midwifery and Allied Health Professions Research Unit, Glasgow Caledonian University, Glasgow, UK), Kathryn VandenBerg (Nursing, Midwifery and Allied Health Professions Research Unit, Glasgow Caledonian University, Glasgow, UK), Linda J. Williams (Usher Institute, University of Edinburgh, Edinburgh, UK), Louise R. Williams (Nursing, Midwifery and Allied Health Professions Research Unit, Glasgow Caledonian University, Glasgow, UK), Masahiro Abo (Department of Rehabilitation Medicine, The Jikei University School of Medicine, Tokyo, Japan), Frank Becker (Institute of Clinical Medicine, Faculty of Medicine, University of Oslo, Oslo, Norway, & Sunnaas Rehabilitation Hospital, Nesoddtangen, Norway), Audrey Bowen (Division of Neuroscience and Psychology, School of Biological Sciences, Faculty of Biology, Medicine and Health, University of Manchester, Manchester Academic Health Science Centre, Manchester, UK), Caitlin Brandenburg (School of Health and Rehabilitation Sciences, The University of Queensland, Brisbane, QLD, Australia), Caterina Breitenstein (Department of Neurology with Institute of Translational Neurology, University of Münster, Münster, Germany), Stefanie Bruehl (St Mauritius Rehabilitation Centre, Meerbusch, Germany; Clinical and Cognitive Neurosciences, Department of Neurology, Medical Faculty, RWTH Aachen University, Aachen, Germany; and Division of Neuroscience and Experimental Psychology, University of Manchester, Manchester, UK), David A. Copland (School of Health and Rehabilitation Sciences, The University of Queensland, Brisbane, QLD, Australia), Tamara B. Cranfill (Special Education, Eastern Kentucky University, Richmond, KY), Marie di Pietro-Bachmann (Division of Neurorehabilitation, Department of Clinical Neurosciences, University Hospitals and University of Geneva, Geneva, Switzerland), Pamela Enderby (School of Health and Related Research [ScHARR], University of Sheffield, Sheffield, UK), Joanne Fillingham (Nursing Directorate, NHS Improvement, London, UK), Federica Lucia Galli (Neurorehabilitation Clinic, Neurological Sciences Department, Marche Polytechnic University, Ospedali Riuniti di Ancona, Ancona, Italy), Marialuisa Gandolfi (Department of Neuroscience, Biomedicine and Movement Sciences, University of Verona, Verona, Italy), Bertrand Glize (Handicap Activity Cognition Health, University of Bordeaux, Bordeaux, France, & Department of Physical Medicine and Rehabilitation, Centre Hospitalier Universitaire [CHU] de Bordeaux, Bordeaux, France), Erin Godecke ( School of Medical and Health Sciences, Edith Cowan University, Perth, WA, Australia), Neil Hawkins (Institute of Health & Wellbeing, University of Glasgow, Glasgow, UK), Katerina Hilari (Division of Language and Communication Science, City, University of London, London, UK), Jacqueline Hinckley (Nova Southeastern University, Fort Lauderdale, FL), Simon Horton (School of Health Sciences, University of East Anglia, Norwich, UK), David Howard (School of Education Communication and Language Sciences, Newcastle University, Newcastle Upon Tyne, UK), Petra Jaecks (Faculty of Linguistics and Literary Studies, Bielefeld University, Bielefeld, Germany), Elizabeth Jefferies (Department of Psychology, University of York, York, UK), Luis M.T. Jesus (School of Health Sciences [ESSUA] and Institute of Electronics and Informatics Engineering of Aveiro [IEETA], University of Aveiro, Aveiro, Portugal), Maria Kambanaros (Department of Rehabilitation Sciences, Cyprus University of Technology, Limassol, Cyprus), Eun Kyoung Kang (Department of Rehabilitation Medicine, Kangwon National University Hospital, Gangwon-do, Republic of Korea), Eman M. Khedr (Department of Neurology, Assiut University Hospital, Assiut, Egypt), Anthony Pak-Hin Kong (School of Communication Sciences and Disorders, University of Central Florida, Orlando, FL), Tarja Kukkonen (Ear, Nose and Throat [ENT]/Department of Phoniatry, Tampere University Hospital, Tampere, Finland), Marina Laganaro (Faculty of Psychology and Educational Science, University of Geneva, Geneva, Switzerland), Matthew A. Lambon Ralph (Medical Research Council Cognition and Brain Sciences Unit, University of Cambridge, Cambridge, UK), Ann Charlotte Laska (Department of Clinical Sciences, Karolinska Institutet, Danderyd Hospital, Stockholm, Sweden), Béatrice Leemann ( Neurorééducation, Département des Neurosciences niques, Hôpitaux Universitaires de Genève, Geneva, Switzerland), Alexander P. Leff (Department of Brain Repair and Rehabilitation, Institute of Neurology, University College London, London, UK), Roxele R. Lima (Department of Speech Language Pathology, Educational Association Bom Jesus – IELUSC, Santa Catarina, Brazil), Antje Lorenz Institut für Psychologie, Humboldt University Berlin, Berlin, Germany), Brian Mac Whinney (Department of Psychology, Carnegie Mellon University, Pittsburgh, PA), Rebecca Shisler Marshall (Department of Communication Sciences and Special Education, University of Georgia, Athens, GA), Flavia Mattioli (Neuropsychology Unit, Azienda Socio Sanitaria Territoriale [ASST] Spedali Civili of Brescia, Brescia, Italy), İlknur Maviş (Department of Speech and Language Therapy, Anadolu University, Eskişehir, Turkey), Marcus Meinzer (UQ Centre for Clinical Research, The University of Queensland, Brisbane, QLD, Australia), Reza Nilipour (Department of Speech Therapy, University of Social Welfare and Rehabilitation Sciences, Tehran, Islamic Republic of Iran), Enrique Noé (NEURORHB-Hospitales Vithas, Valencia, Spain), Nam-Jong Paik (Department of Rehabilitation Medicine, Seoul National University College of Medicine, Seoul National University Bundang Hospital, Seongnam, Republic of Korea), Rebecca Palmer (School of Health and Related Research [ScHARR], University of Sheffield, Sheffield, UK), Ilias Papathanasiou (Department of Speech and Language Therapy, Technological Educational Institute of Western Greece, Patras, Greece), Brigida F Patricio (Speech Therapy Department of Health School of Polytechnic Institute of Porto, Porto, Portugal), Isabel Pavão Martins (Laboratório de Estudos de Linguagem, Faculdade de Medicina de Lisboa, Universidade de Lisboa, Lisbon, Portugal), Cathy Price (Wellcome Centre for Human Neuroimaging, University College London, London, UK), Tatjana Prizl Jakovac (Department of Speech and Language Pathology, Faculty of Education and Rehabilitation Sciences, University of Zagreb, Zagreb, Croatia), Elizabeth Rochon (Department of Speech–Language Pathology and Rehabilitation Sciences Institute, University of Toronto, Toronto, ON, Canada & Toronto Rehabilitation Institute, Toronto, ON, Canada), Miranda L Rose (School of Allied Health, La Trobe University, Melbourne, VIC, Australia), Charlotte Rosso (Institut du Cerveau et del la Moelle épinière, Sorbonne University, Paris, France & APHP, Urgences Cérébro- Vasculaires, Hôpital de la Pitié Salpêtrière, Paris, France), Ilona Rubi-Fessen (RehaNova Rehabilitation Hospital and Department of Special Education and Rehabilitation, University of Cologne, Cologne, Germany), Marina B. Ruiter (Sint Maartenskliniek, Rehabilitation Centre and Centre for Language Studies, Radboud University, Nijmegen, the Netherlands), Claerwen Snell (Warrington Hospital, Warrington and Halton NHS Foundation Trust, Warrington, UK), Benjamin Stahl (Department of Neurology, Charité Universitätsmedizin Berlin, Berlin, Germany), Jerzy P. Szaflarski (UAB Epilepsy Centre, Department of Neurology, University of Alabama at Birmingham, Birmingham, AL), Shirley A. Thomas (Division of Rehabilitation, Ageing and Wellbeing, School of Medicine, University of Nottingham, Nottingham, UK), Mieke van de Sandt-Koenderman (Rijndam Rehabilitation, Rotterdam, the Netherlands; Department of Rehabilitation Medicine, Erasmus MC, University Medical Center Rotterdam, the Netherlands), Ineke van der Meulen (Rijndam Rehabilitation, Rotterdam, the Netherlands; Department of Rehabilitation Medicine, Erasmus MC, University Medical Center Rotterdam, the Netherlands), Evy Visch-Brink (Department of Neurology and Neurosurgery, Erasmus University Medical Center, Rotterdam, the Netherlands), Linda Worrall (School of Health and Rehabilitation Sciences, The University of Queensland, Brisbane, QLD, Australia), Heather Harris Wright (College of Allied Health Sciences, North Carolina University, Greenville, SC), Marian C. Brady (Nursing, Midwifery and Allied Health Professions Research Unit, Glasgow Caledonian Un University, Glasgow, UK).
