# Supplementary material for: Predictors of Poststroke Aphasia Recovery: A Systematic Review-Informed Individual Participant Data Meta-Analysis
Source: Stroke. 2021 Mar 15;52(5):1778–87. doi: 10.1161/STROKEAHA.120.031162 (PMC8078126; doi:10.1161/STROKEAHA.120.031162)
Supplement: Supplementary file 1 [file str-52-1778-s001.pdf]

Supplemental Materials

Demographics of post-stroke aphasia recovery: a systematic review-informed individual participant data (IPD) meta-analysis

Supplement 1) Table I: Standardised measures for each language domain

Language domain	Standardised Measurement	Range
Overall Language Ability	Western Aphasia Battery (Aphasia Quotient)	0-100
Auditory Comprehension	Aachen Aphasia Test Token Test	0-50 (reflecting positive scoring)
Naming	Boston Naming Test	0-60
Reading	Reading subtest of the Comprehensive Aphasia Test	0-74
Writing	Writing subtest of the Comprehensive Aphasia Test	0-76
Other Spoken Language	Porch Index of Communicative Ability	0-16
Functional Communication	Aachen Aphasia Test Spontaneous Communication domain	0-5

Supplement 2) Table II: Participant Demography

Parameter		Language Domain			
		**Overall language ability N=943**	**Auditory comprehension N=1056**	**Naming N=791**	**Functional Communication N=974**
**Age, years (median, IQR)**		63 (53-73), n=914	61 (51-69), n=1046	61 (52-69)	67 (57-77)
**By age group**	≤55 years<br>56-65 years<br>66-75 years<br>>75 years<br>Missing	281 (31%)<br>248 (27%)<br>195 (21%)<br>190 (21%)<br>29	360 (34%)<br>322 (31%)<br>231 (22%)<br>133 (13%)<br>10	269 (34%)<br>245 (31%)<br>190 (24%)<br>87 (11%)<br>0	208 (21<br>228 (23<br>267 (27<br>271 (28<br>0
**Time since stroke, days (median, IQR)**		36 (7-548), n=940	116 (18-883), n=1046	131 (30-883)	28 (6-244), n=962
**By Time group**	0-28 days<br>4 weeks-3 months<br>3-6 months<br>>6 months<br>Missing	442 (47%)<br>107 (11%)<br>59 (6%)<br>332 (35%)<br>3	339 (32%)<br>151 (14%)<br>97 (9%)<br>459 (44%)<br>10	194 (25%)<br>143 (18%)<br>106 (13%)<br>348 (44%)<br>0	501 (52<br>129 (13<br>69 (7<br>263 (27<br>12
**Sex**	Female<br>Male<br>Missing	360 (40%)<br>544 (60%)<br>39	385 (37%)<br>660 (63%)<br>11	278 (36%)<br>502 (64%)<br>11	417 (43<br>557 (57<br>0
**Ethnicity**	Asian<br>Black<br>White<br>Not specified	24 (2.5%)<br>0<br>25 (2.5%)<br>894 (95%)	22 (2%)<br>4 (0.4%)<br>71 (7%)<br>959 (91%)	0<br>5 (0.6%)<br>72 (9%)<br>714 (90%)	0<br>5 (0.5<br>80 (8<br>889 (91
**Handed**	Right<br>Left<br>Ambidextrous<br>Missing	616 (95%)<br>25 (5%)<br>7 (1%)<br>295	852 (95%)<br>37 (4%)<br>12 (1%)<br>155	586 (94%)<br>29 (5%)<br>8 (1%)<br>168	536 (94<br>24 (4<br>10 (2<br>404
**Hemisphere**	Left<br>Right<br>Bilateral	627 (90%)<br>55 (8%)	725 (90%)<br>61 (8%)<br>20 (2%)	618 (89%)<br>59 (9%)	645 (98<br>11 (2<br>5 (0.7

	Missing	18 (3%)<br>243	250	16 (2%)<br>98	313
**Type**	ICH<br>Ischaemic<br>Mixed<br>SAH<br>Unclassified<br>Missing	90 (10%)<br>628<br>(68%)<br>29 (3%)<br>14 (1.5%)<br>162<br>(18%)<br>20	128 (12%)<br>671 (64%)<br>0<br>15 (1%)<br>227 (22%)<br>15	76<br>(10%)<br>502<br>(64%)<br>0<br>8 (1%)<br>201<br>(26%)<br>4	70 (7<br>704 (74<br>0<br>9 (1<br>164 (17<br>19
**Living context**	Alone<br>Formal care<br>Living with others<br>Missing	55 (34%)<br>7 (4%)<br>102<br>(62%)<br>776	19 (18%)<br>30 (29%)<br>55 (53%)<br>952	23<br>(21%)<br>30<br>(27%)<br>58<br>(52%)<br>680	86 (34<br>30 (12<br>136 (54<br>721
**Language**	Arabic<br>Danish<br>Dutch<br>English<br>Finnish<br>French<br>German<br>Greek<br>Italian<br>Japanese<br>Korean<br>Norwegian<br>Portuguese<br>Spanish<br>Swedish	29 (3%)<br>19 (2%)<br>0<br>329<br>(35%)<br>36 (4%)<br>99 (11%)<br>128<br>(14%)<br>0<br>0<br>24 (3%)<br>21 (2%)<br>39 (4%)<br>25 (3%)<br>0<br>194<br>(21%)	0<br>0<br>292 (28%)<br>278 (26%)<br>31 (3%)<br>0<br>175 (17%)<br>0<br>115 (11%)<br>58 (5%)<br>21 (2%)<br>39 (4%)<br>25 (2%)<br>0<br>22 (2%)	0<br>0<br>172<br>(22%)<br>309<br>(39%)<br>36 (5%)<br>0<br>42 (5%)<br>38 (5%)<br>50 (6%)<br>36 (5%)<br>21 (3%)<br>39 (5%)<br>25 (3%)<br>10 (1%)<br>13 (2%)	0<br>19 (2%)<br>171 (18%)<br>357 (37%)<br>23 (2%)<br>0<br>112 (12%)<br>38 (4%)<br>0<br>0<br>0<br>0<br>23 (2%)<br>0<br>231 (24%)

Supplement 3) Figure I: Transformed language domain scores, stratified by contributing assessment tool

a) Overall-language-ability

Overall Language Ability: Standardised scores stratified by assessment tools used

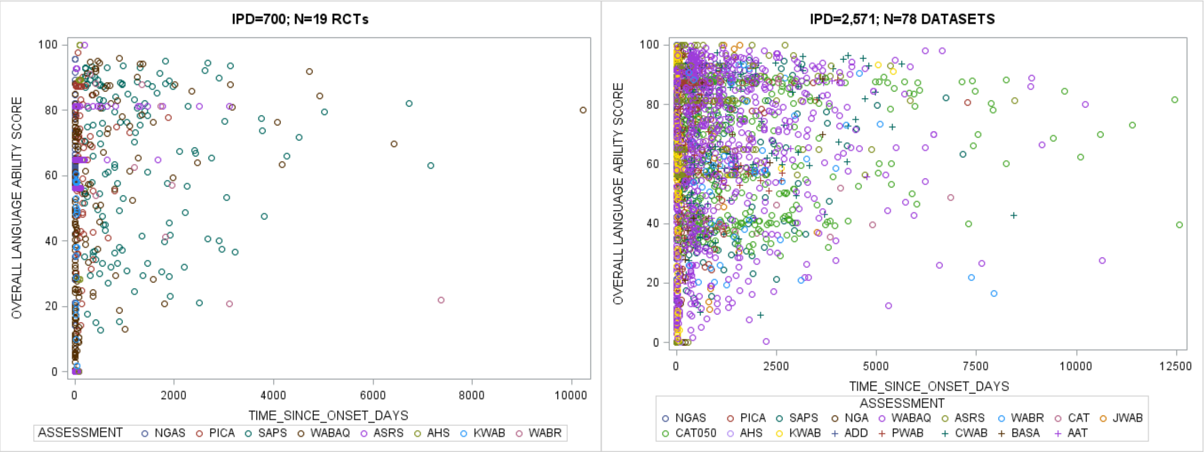

b) Auditory comprehension

Auditory comprehension: Standardised scores stratified by assessment tools used

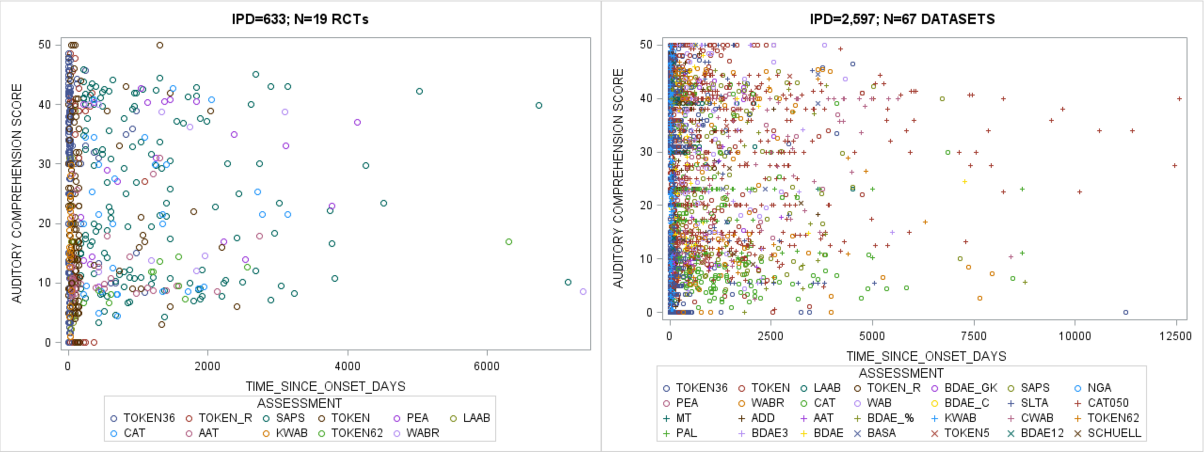

c) Naming

Naming: Standardised scores stratified by assessment tools used

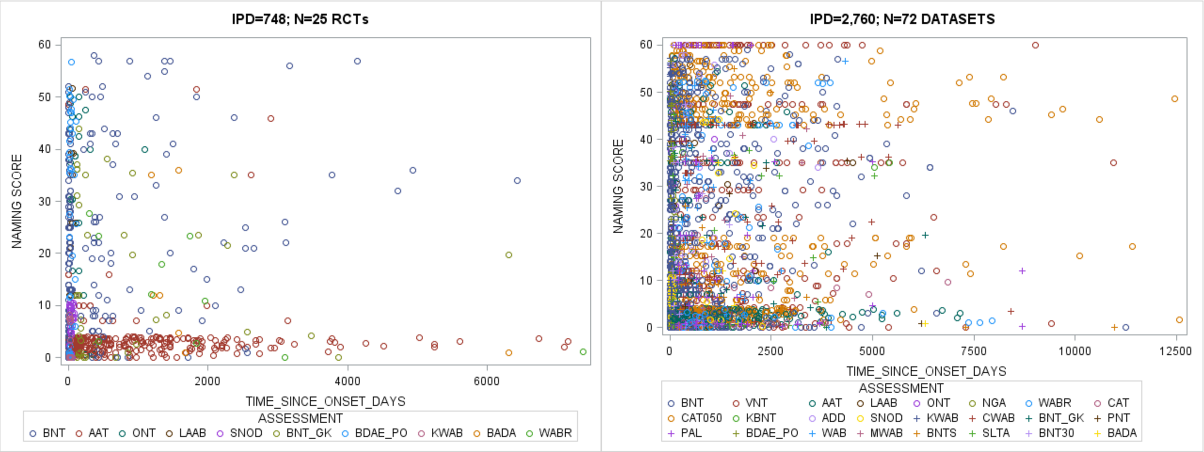

d) Functional communication: observer-rated activity

Functional Communication: Standardised scores stratified by assessment tools used

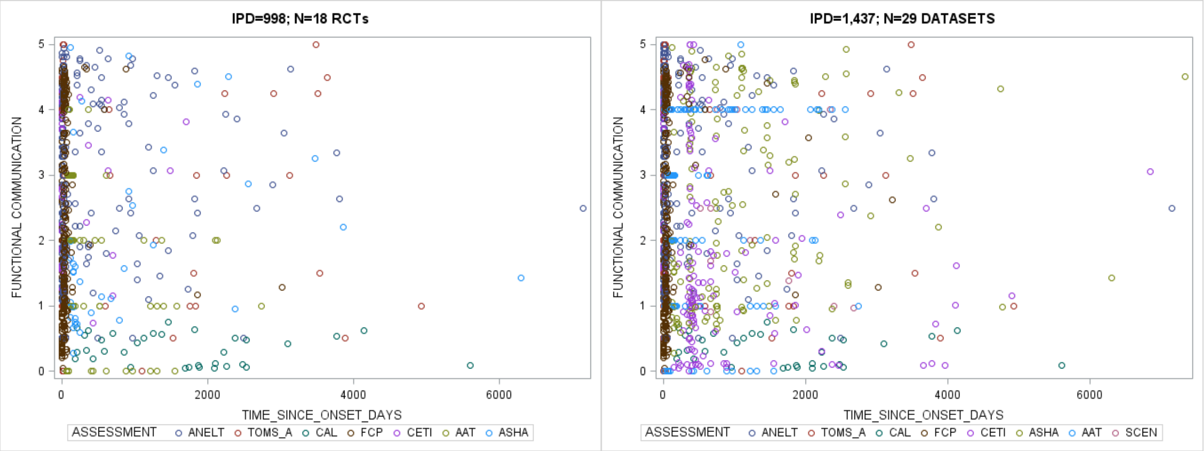

The spread of data points overlapped, with no clustering of transformed scores from contributing assessment tools. The transformed values generated by each of the assessment tools were therefore considered to be valid and fell within an expected range.

Supplement 4a) Table III: Associations between demographic factors and overall language ability (WAB-AQ)

Variable	RCTs	IPD*	Estimate of means	Lower Quartile	Upper Quartile	All Studies Designs	IPD	Estimate of means	Lower Quartile	Upper Quartile
**Female**	11	206	14.25	9.01	19.50	16	250	14.15	9.66	18.65
**Male**	11	276	12.30	7.16	17.44	17	378	12.17	7.89	16.46
**<55 years**	11	136	15.43	9.95	20.91	17	192	16.51	10.03	22.99
**56-65 years**	11	141	12.36	6.90	17.82	16	178	12.88	6.38	19.38
**66-75**	10	96	11.49	5.73	17.25	14	123	13.22	6.69	19.75
**>75**	7	109	13.81	7.82	19.81	9	135	13.83	6.94	20.73
**0-28 days**	8	260	19.14	13.89	24.39	9	335	20.99	13.78	28.20
**1-3 months**	6	64	16.17	10.20	22.13	8	71	16.44	9.02	23.86
**3-6 months**	3	16	9.60	0.07	19.14	5	19	9.58	-0.07	19.22
**>6 months**	4	142	8.19	-0.12	16.50	8	203	9.44	2.81	16.07
					Random (no)	6	146	14.17	7.36	20.99
					Random (yes)	11	482	12.37	7.70	17.04

*Individual participant data

Supplement 4b) Table IV: Associations between demographic factors and auditory comprehension (AAT-TT)

Variable	RCTs	IPD*	Estimate of means	Lower Quartile	Upper Quartile	All Study Designs	IPD	Estimate of means	Lower Quartile	Upper Quartile
**Female**	16	211	3.82	0.98	6.66	22	239	4.92	2.15	7.70
**Male**	16	329	3.10	0.35	5.85	21	402	4.35	1.65	7.04
**<55 years**	16	178	6.05	3.16	8.94	22	232	7.05	4.32	9.77
**56-65 years**	16	182	2.83	-0.04	5.71	22	212	4.31	1.52	7.09
**66-75**	15	116	3.50	0.46	6.53	19	131	4.61	1.65	7.58
**>75**	12	64	1.46	-1.90	4.82	14	66	2.57	-0.74	5.88
**0-28 days**	6	139	5.25	1.69	8.82	6	139	6.33	2.57	10.08
**1-3 months**	9	97	4.27	1.07	7.47	10	106	5.16	1.99	8.34
**3-6 months**	6	61	2.88	-1.81	7.56	8	65	4.32	0.04	8.60
**>6 months**	9	243	1.44	-1.85	4.72	15	331	2.73	0.12	5.34
					Random (no)	5	101	6.11	1.65	10.57
					Random (yes)	16	540	3.16	0.67	5.65

*Individual participant data

Supplement 4c) Table V: Associations between demographic factors and naming (BNT)

Variable	RCTs	IPD*	Estimate of means	Lower Quartile	Upper Quartile	All Study Designs	IPD	Estimate of means	Lower Quartile	Upper Quartile
**Female**	13	165	6.92	2.47	11.36	21	219	7.54	3.84	11.23
**Male**	13	220	6.70	2.33	11.07	21	333	7.73	4.10	11.36
**<55 years**	13	103	9.31	4.71	13.90	21	181	9.72	6.00	13.44
**56-65 years**	13	124	7.34	2.79	11.90	21	169	8.08	4.30	11.87
**66-75**	12	97	6.16	1.48	10.84	18	134	7.35	3.48	11.22
**>75**	11	61	4.42	-0.56	9.40	15	68	5.38	1.11	9.66
**0-28 days**	5	129	11.12	5.71	16.54	6	138	11.35	6.62	16.09
**1-3 months**	8	93	7.74	2.89	12.59	10	116	8.70	4.56	12.84
**3-6 months**	6	70	4.26	-1.58	10.10	9	92	5.70	1.17	10.23
**>6 months**	7	93	4.11	-1.08	9.29	15	206	4.78	1.08	8.48
					Random (no)	8	167	8.56	2.88	14.24
					Random (yes)	13	385	6.71	2.57	10.85

*Individual participant data

Supplement 4d) Table VI: Associations between demographic factors and functional communication (AAT-SC)

Variable	RCTs	IPD*	Estimate of means	Lower Quartile	Upper Quartile	All Study Designs	IPD	Estimate of means	Lower Quartile	Upper Quartile
**Female**	14	236	0.76	0.48	1.03	18	275	0.69	0.40	0.98
**Male**	14	296	0.57	0.30	0.84	19	372	0.54	0.26	0.83
**<55 years**	14	147	0.75	0.46	1.04	19	181	0.69	0.39	0.99
**56-65 years**	13	145	0.70	0.41	1.00	18	170	0.66	0.36	0.96
**66-75**	14	121	0.55	0.25	0.85	18	148	0.52	0.21	0.82
**>75**	12	119	0.65	0.34	0.96	15	148	0.61	0.30	0.92
**0-28 days**	6	232	1.05	0.70	1.40	7	277	0.97	0.61	1.34
**1-3 months**	5	68	0.87	0.51	1.23	6	70	0.83	0.46	1.21
**3-6 months**	4	62	0.40	-0.06	0.87	5	63	0.37	-0.10	0.83
**>6 months**	7	170	0.33	0.00	0.66	11	237	0.30	0.01	0.60
					Random (no)	5	115	0.57	0.11	1.03
					Random (yes)	14	532	0.67	0.41	0.94

*Individual participant data

Supplement 5) Figure II: Absolute and Relative Proportion of Change from Baseline in Language Domain Scores across All Study Designs

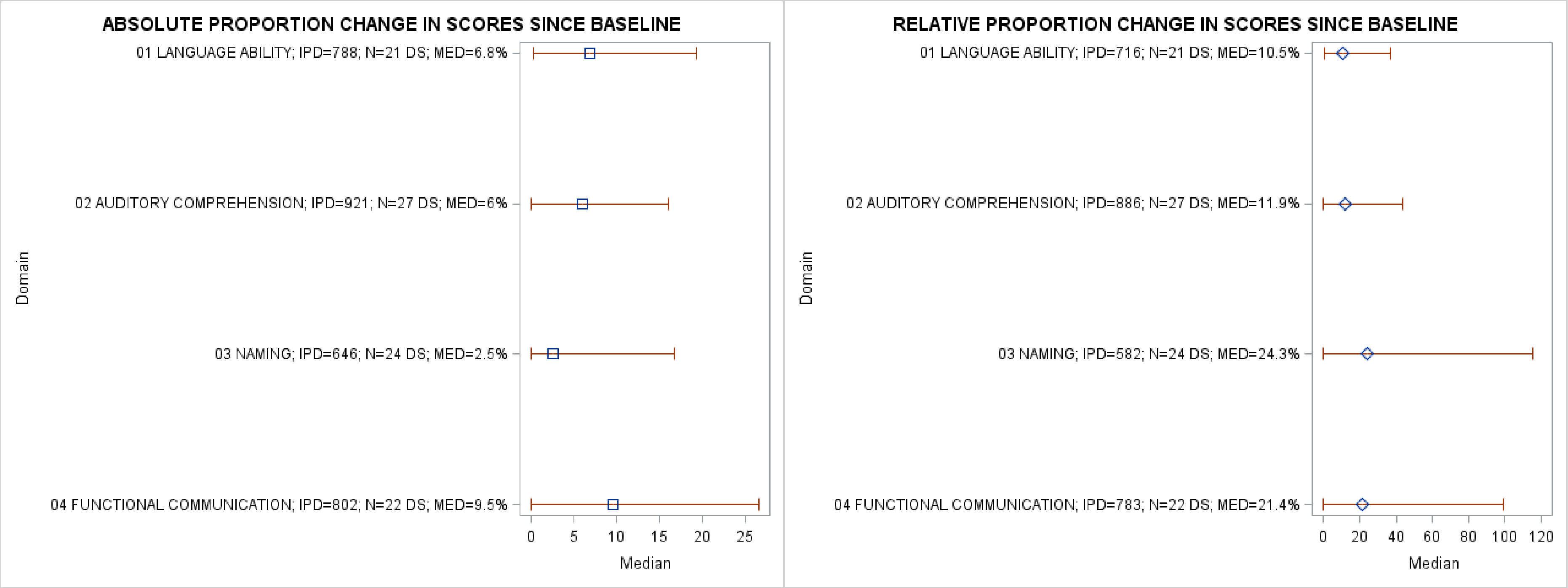

Key: IPD= Individual Participant Data; N=number of datasets; MED=median proportion of recovery
